# Contrast-enhanced ultrasound-guided liver biopsy with flow cytometry analysis for the diagnosis of secondary hemophagocytic syndrome: A case report

**DOI:** 10.1097/MD.0000000000048256

**Published:** 2026-04-10

**Authors:** Huihui Ma, Qing Pan, Xin Jin, Shuyuan Tian

**Affiliations:** a College of Integrated Traditional Chinese and Western Clinical Medicine, Tongde Hospital of Zhejiang Province Affiliated to Zhejiang Chinese Medical University, Hangzhou, Zhejiang, China; b Department of Pathology, Tongde Hospital of Zhejiang Province Afflicted to Zhejiang Chinese Medical University, Hangzhou, Zhejiang, China; c Department of Clinical Laboratory, Tongde Hospital of Zhejiang Province Afflicted to Zhejiang Chinese Medical University, Hangzhou, Zhejiang, China; d Department of Ultrasound, Tongde Hospital of Zhejiang Province Afflicted to Zhejiang Chinese Medical University, Hangzhou, Zhejiang, China.

**Keywords:** contrast, enhanced ultrasound, flow cytometry, guided liver biopsy, secondary hemophagocytic syndrome

## Abstract

**Rationale:**

Hemophagocytic lymphohistiocytosis (HLH) is a hereditary or acquired hyperinflammatory response syndrome. Diagnosis of HLH is challenging.

**Patient concerns:**

We report a case of a 41-year-old male who was admitted to the hospital due to recurrent fever for 1 month and hematochezia for 2 days.

**Diagnoses:**

Positron emission tomography/computed tomography (PET/CT) showed multiple intrahepatic high-uptake nodules, and B-mode ultrasound could not accurately localize them. Contrast-enhanced ultrasound (CEUS)-guided liver biopsy combined with flow cytometry confirmed the final diagnosis of natural killer (NK)/T-cell lymphoma-associated hemophagocytic syndrome. Pathological confirmation was made with a lymph node biopsy.

**Interventions:**

The patient received the gemcitabine-dexamethasone-cisplatin (GDP) regimen and symptomatic supportive treatments.

**Outcomes:**

Unfortunately, the patient experienced persistent disease progression and passed away.

**Lessons:**

HLH is a hyperinflammatory response syndrome caused by immune dysfunction. Lymphoma-associated hemophagocytic syndrome is the most common type of HLH. Diagnosis can be confirmed through CEUS-guided biopsy, flow cytometry analysis, and relevant clinical information.

## 1. Introduction

Hemophagocytic syndrome (HPS), or hemophagocytic lymphohistiocytosis (HLH), is an excessive inflammatory response syndrome. It arises from a hereditary or acquired immunoregulatory abnormality and is characterized by the massive activation, proliferation, and release of inflammatory factors from lymphocytes. The diagnosis of HLH is challenging to make. Here, we report a case of HLH secondary to natural killer (NK)/T-cell lymphoma. The diagnosis was confirmed by contrast-enhanced ultrasound (CEUS)-guided liver biopsy with flow cytometry (FCM). While FCM can provide immunophenotypic information suitable for hematologic disease classification, its application is typically limited to peripheral blood and bone marrow samples. Meanwhile, liver biopsy specimens are commonly used for conventional histopathological analysis. Therefore, we applied FCM to a CEUS-guided liver biopsy specimen. Ultimately, this case report highlights the value of CEUS compared with conventional ultrasound for biopsy and the diagnostic value of FCM in lymphoma-associated HLH.

## 2. Case presentation

### 2.1. Clinical data

A 41-year-old man with a history of hypertension and chronic hepatitis B was admitted to the hospital with recurrent fever (peak 38.7°C) for 1 month and hematochezia for 2 days. A previous blood test showed a leukocyte count of 3.0 × 10^9^/L, with the remaining 2 lines normal and HP (+). HP-associated leukopenia was considered, and the patient received anti-HP and leukocyte-increasing treatment (Leucogen; Jiangsu Jibeier Pharmaceutical Co., Ltd., Zhejiang, China). Two days later, the patient developed melena and pancytopenia. On admission, the patient’s laboratory findings were as follows: hemoglobin 68.0 g/L, erythrocytes 2.49 × 10^12^/L, leukocytes 1.0 × 10^9^/L, platelets 73 × 10^9^/L; aspartate aminotransferase (AST) 152 U/L, alanine aminotransferase (ALT) 117 U/L, albumin 21.7 g/L; prothrombin time (PT) 13.7 seconds, partial thromboplastin time (APTT) 32.5 seconds, d-dimer 0.74 mg/L; interleukin-6 (IL-6) 7.2 pg/mL, interleukin-10 (IL-10) 309.9 pg/mL, interferon-gamma (IFN-γ) 1207.6 pg/mL. Bone marrow biopsy revealed a moderate number of megakaryocytes, impaired platelet production, and no abnormal cells.

### 2.2. Imaging examinations

**Figure 1.**
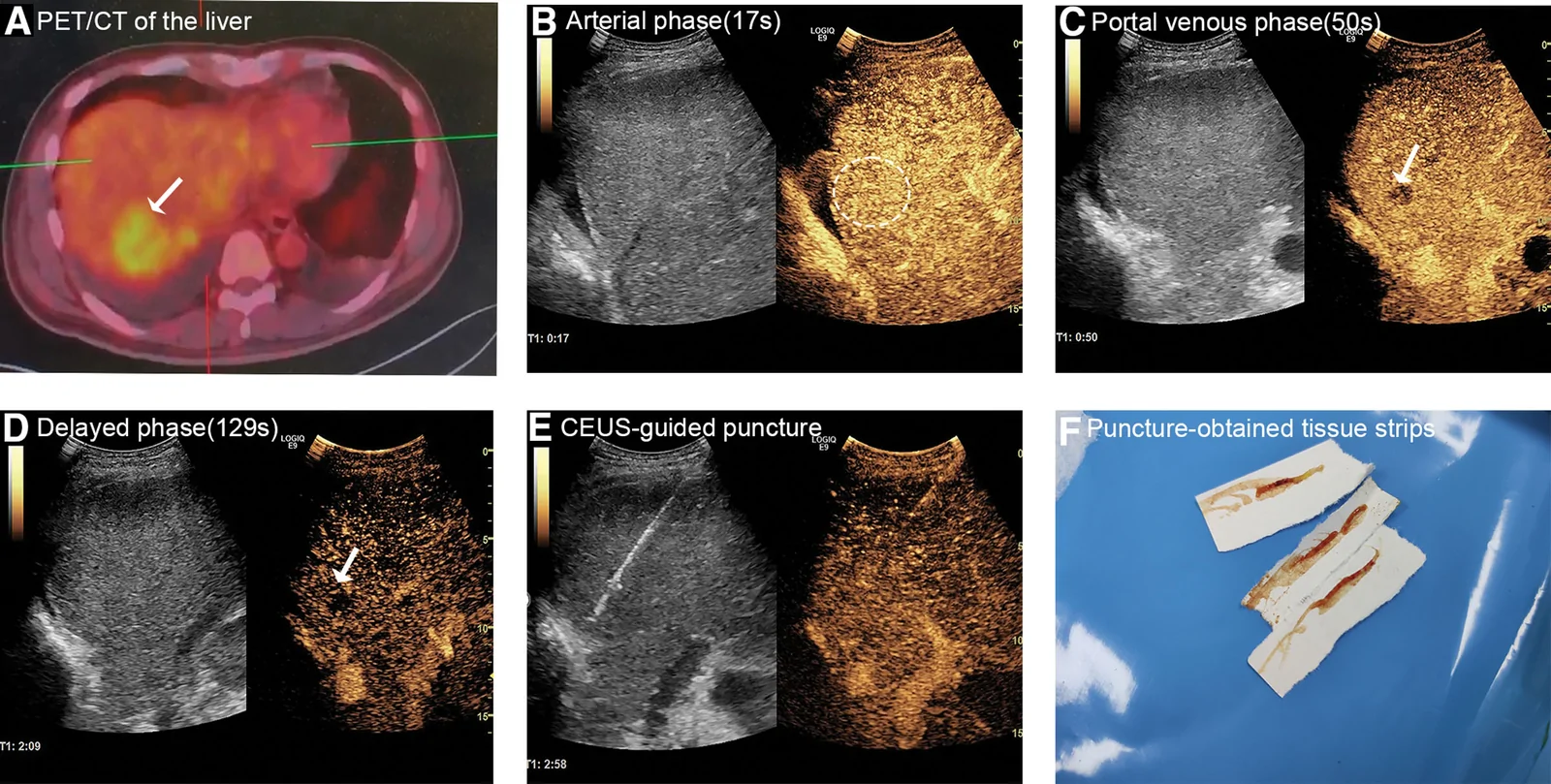
Liver lesion imaging and biopsy in a 41-year-old man with recurrent fever and melena. (A) PET-CT images showed liver uptake of FDG (arrow), suggesting multiple hepatic lesions suspicious for malignancy. (B–D) CEUS was performed with injection of 2.4 mL sulfur hexafluoride microbubbles (SonoVue; Bracco) at a low mechanical index (<0.12): (b) Arterial phase (17 seconds): the lesion showed iso-enhancement (circle); (C) Portal venous phase (50 seconds): the lesion showed hypo-enhancement (arrow); (D) Delayed phase (129 seconds): the lesion showed hypo-enhancement (arrow); (E) CEUS-GB was performed with an 18G automatic cutting biopsy needle after clear visualization of the lesion. (F) Three tissue strips were obtained and submitted for pathological and flow cytometry analysis. The patient was diagnosed with NK/T-cell lymphoma and hemophagocytic syndrome. CEUS = contrast-enhanced ultrasound; CEUS-GB = contrast-enhanced ultrasound-guided biopsy, FDG = 18F-fluorodeoxyglucose, PET-CT = positron emission tomography.

Positron emission tomography/computed tomography (PET/CT) images showed multiple 18F-fluorodeoxyglucose high-uptake areas in the liver (Fig. 1A) and in various other areas, including in the enlarged abdominal lymph nodes and multiple areas in the bone marrow, as well as small nodes in the left subclavian, right sixth to seventh intercostal space, and left internal breast area. The findings were highly suggestive of lymphoma. Thus, a puncture biopsy of the intrahepatic lesion or superficial lymph nodes was recommended.

### 2.3. Ultrasound findings

After admission, the patient developed hypovolemic shock and initially received symptomatic supportive therapy. To further investigate the etiology, a puncture biopsy of the intrahepatic lesion was performed. To improve the biopsy success rate, conventional ultrasound-guided biopsy (US-GB) was considered. However, B-mode ultrasound showed hepatomegaly but no intrahepatic lesions. Thus, CEUS was performed with the injection of 2.4 mL sulfur hexafluoride microbubbles (SonoVue; Bracco, Milan, Italy). Real-time contrast-enhanced imaging was performed at a low mechanical index (<0.12). The target lesion, undetected on B-mode ultrasound, showed iso-enhancement in the arterial phase (Fig. 1B) and hypo-enhancement in the venous and delayed phases (Fig. 1C, D).

When the lesion was clearly visualized, an 18-G automatic cutting biopsy needle was advanced to its edge. The needle was activated 3 times, yielding 3 tissue strips in total (Fig. 1E, F). No tract embolization was used. The patient was kept on bed rest for 4 hours for close observation. The procedure was completed without bleeding complications; however, prebiopsy bleeding risk assessment remains necessary when performing this procedure.^[1]^ In our center, invasive tissue biopsies in HLH patients with coagulopathy are performed when the platelet count is ≥50 × 10^9^/L and the international normalized ratio (INR) ≤ 1.5, in accordance with standards for high bleeding-risk image-guided procedures.^[1]^

### 2.4. Pathological and FCM findings

**Figure 2.**
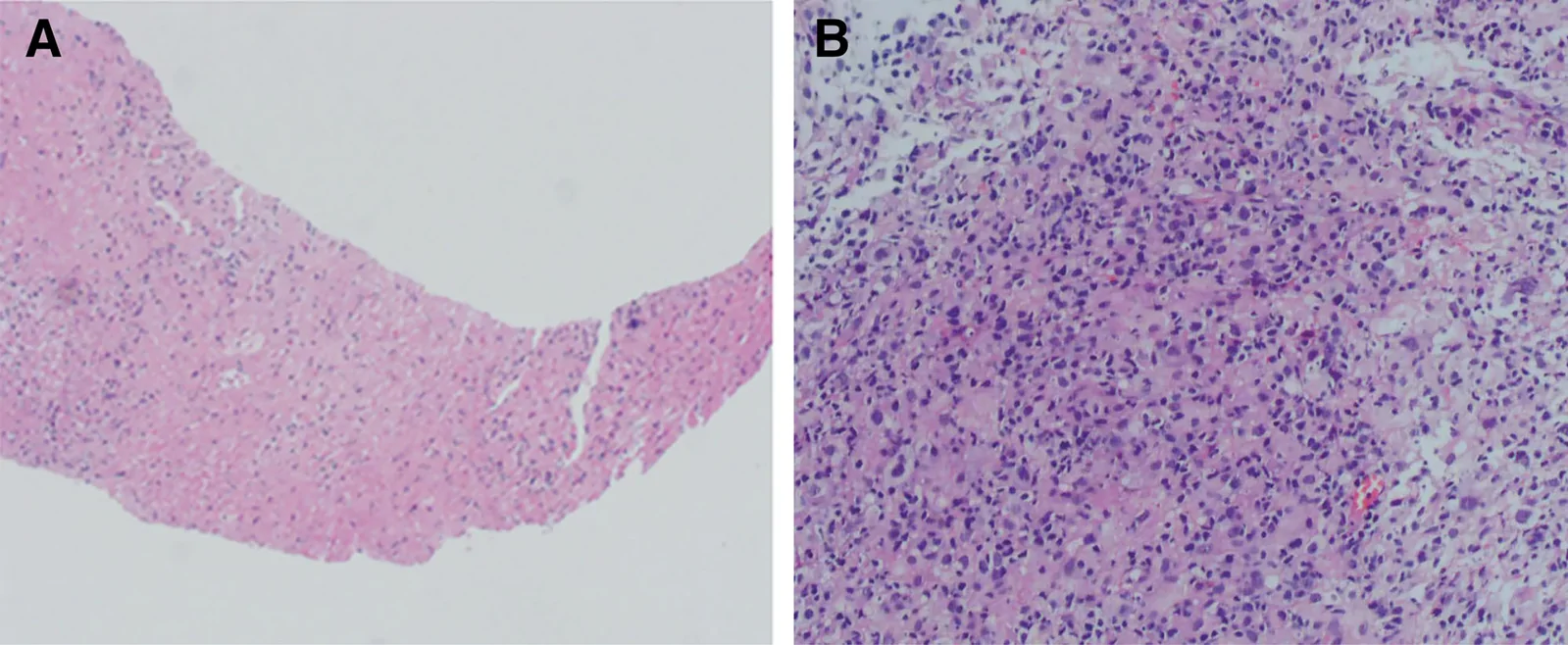
The characteristics of pathology IHC. (A) Liver pathologic analysis revealed extensive necrotic hepatic tissues were interspersed with scattered atypical lymphocytes. (B) Lymph node pathologic analysis revealed unclear lymph node structure with lymphoid tissue hyperplasia and atypia (Hematoxylin and Eosin staining, magnification ×100). The pathological diagnoses all indicated abnormal lymphocytic infiltration. Based on clinical presentation, lymphoid hematopoietic system tumors were considered. IHC = immunohistochemistry.

Tissue obtained from the CEUS-GB was subjected to conventional pathology and FCM, respectively. Pathological examination of the liver specimen revealed extensive necrotic tissue with scattered heterogeneous lymphocytes, suggestive of lymphohematopoietic malignancy. The specific tumor type was uncertain (Fig. 2A).

**Figure 3.**
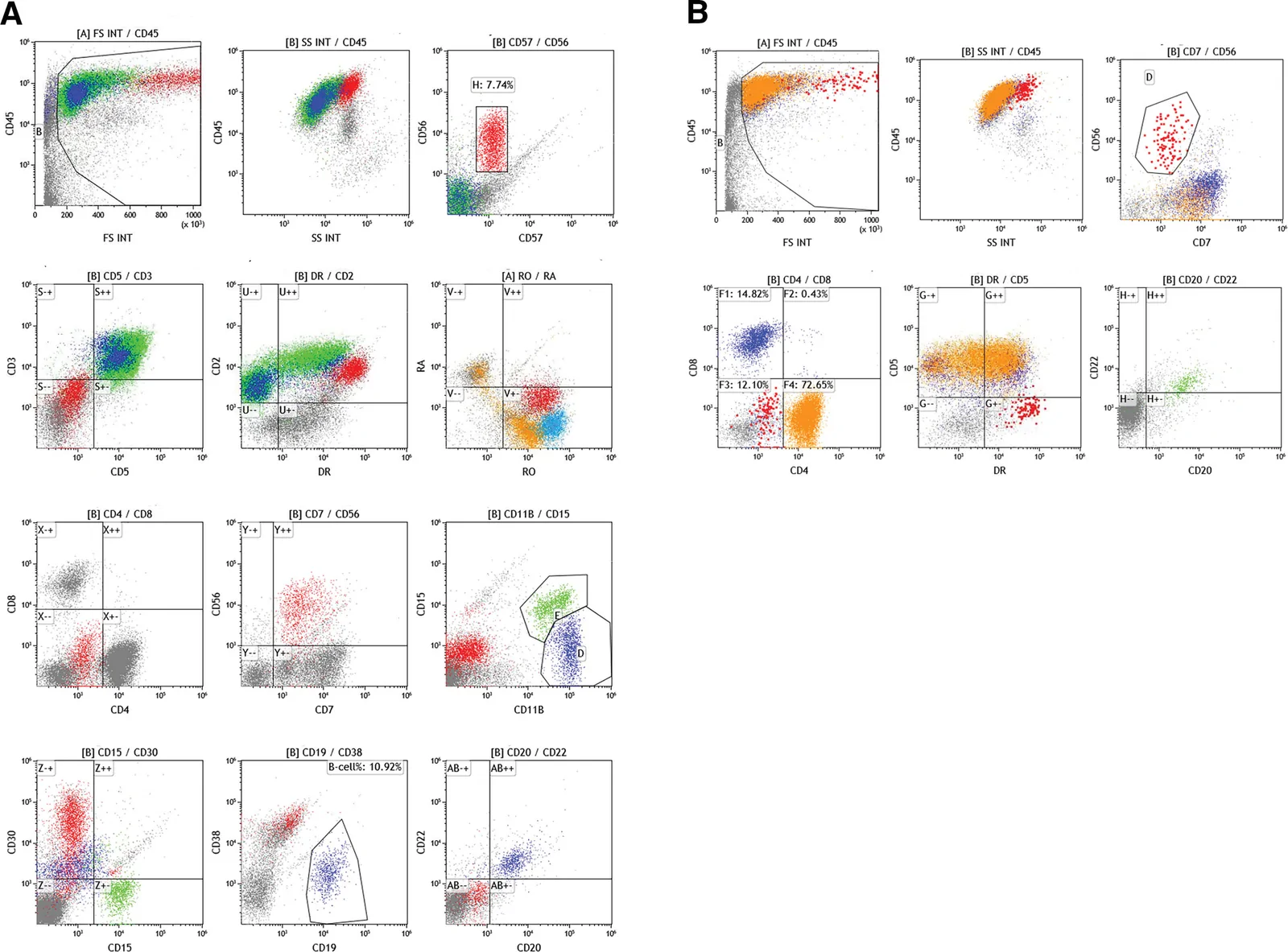
The flow cytometry analysis of biopsy tissue was performed on a Navios flow cytometer (Beckman Coulter). (A) The flow cytometry analysis of the liver biopsy tissue revealed an abnormal cell population (about 7.74%). The cells were CD45, CD56, CD2, CD30, CD7, CD38, CD45RO, HLA-DR (bright) positive. (B) The FCM of lymph node biopsy tissue revealed an abnormal cell population (about 0.68%). The cells were positive for CD38, CD56, and HLA-Dr Their antigen expression and FCM immunophenotyping results supported a diagnosis of NK/T-cell lymphoma. CD = cluster of differentiation, FCM = flow cytometry, HLA-Dr = human leukocyte antigen-D related, NK/T-cell lymphoma = natural killer/T-cell lymphoma.

Flow cytometry analysis was then performed using a Navios flow cytometer (Beckman Coulter, Inc., Brea). The following primary antibodies and isotype-matched antibodies were obtained from Beckman Coulter and Bio Legend: Pacific Blue-conjugated anti-CD20 (HRC20), FITC-conjugated anti-CD45RA (ALB11), PE-conjugated anti-CD45RO (UCHL1), ECD-conjugated anti-CD5 (BL1a), PE/Cy5.5-conjugated anti-CD56 (N901). For each sample, a minimum of 1,00,000 events were acquired. The procedure was performed as follows: propidium iodide (PI) was used to remove dead cells and adherent cells; SS/CD45 gates were used to circle lymphocytes; CD56 was used as a NK cell marker, and CD3 was used for T cells. Abnormal cells accounted for 7.74% of all cells. These cells showed predominant expression of the antigens CD56, CD2, CD30, CD7 (dim), CD38 (bright), CD45RO, and DR (bright), suggesting NK-cell lymphoma (Fig. 3).

### 2.5. Intervention and outcome

Through consideration of both clinical information and the immunohistochemistry results of subsequent neck lymph node puncture specimens (Fig. 2B), the patient was eventually diagnosed with NK/T-cell lymphoma (stage IVB; the prognostic index for NK cell lymphoma-Epstein-Barr virus was 4 [PINK-E 4], high risk) and hemophagocytic syndrome.

After 2 weeks of gemcitabine-dexamethasone-cisplatin (GDP) treatment, the patient exhibited a slight recovery. Two months later, the patient again developed recurrent fever and elevated cytokine levels, suggestive of HLH relapse. The treatment was adjusted (etoposide 120 mg, d1-2 + sintilimab 200 mg, d1 + dexamethasone 10 mg, d1 + doxorubicin 40 mg, d2 + ruxolitinib 5 mg bid), with poor efficacy. The patient experienced persistent disease progression and developed multisystem organ failure. To reduce suffering, the family opted for palliative care. The patient eventually passed away at home.

## 3. Discussion

Hemophagocytic lymphohistiocytosis is a rare, highly fatal, and rapidly progressive disease that can be divided into 2 types: primary and secondary.^[2]^ Secondary HLH commonly occurs in malignant tumors, infections, and rheumatic diseases. Lymphoma-associated HLH is the most common type of tumor-associated HLH.^[3]^ HLH diagnosis is primarily based on the HLH-2004 guidelines.^[2]^ Here, the patient exhibited typical clinical manifestations (persistent fever, hepatosplenomegaly, cytopenia, hypofibrinogenemia, and hyperferritinemia), meeting the clinical diagnostic criteria for the disease. Pathological examination and FCM of CEUS-guided biopsy specimens confirmed the final diagnosis of HLH secondary to lymphoma.

However, etiological diagnosis remains challenging when a bone marrow biopsy is nondiagnostic, and PET/CT-indicated extramedullary lesions cannot be localized by conventional ultrasound. In this context, the combination of CEUS and FCM offers promise: CEUS-GB can be used to acquire viable tissue, and FCM enables definitive immunophenotypic analysis.

Hepatomegaly is the most common but nonspecific sonographic finding in HLH.^[4]^ In this case, B-mode ultrasound failed to detect the intrahepatic lesions observed on PET/CT. The lesions were isoechoic, likely influencing their identification on conventional ultrasound. For uncertain small lesions, US-GB tends to sample necrotic tissues, while CEUS can distinguish focal lesions from normal liver parenchyma.^[5,6]^ Several studies have shown that combining CEUS and CT can improve the diagnostic sensitivity of liver metastases.^[5]^ In this context, CEUS combined with PET/CT demonstrates good synergy. Specifically, PET/CT can be used to initially localize lesions through whole-body metabolic imaging, then CEUS can be used to dynamically display the extent of the tumor through real-time hemodynamic assessment. As such, CEUS-GB was able to precisely target metabolically active regions in this case. Viable tissue for accurate FCM specimens was obtained with 3 needle passes. Fewer punctures directly reduce vascular injury risk and avoid postoperative complications such as hemorrhage. In summary, PET/CT and CEUS play a complementary role when a conventional ultrasound shows poor results.^[7]^

Hemophagocytes in the bone marrow smears of HLH patients provide the most intuitive diagnostic evidence. However, their absence cannot exclude HLH. Sampling the liver and other affected sites can provide further evidence.^[2,8,9]^ A study of 30 HLH patients complicated with hepatic dysfunction showed hematophagocytosis in all liver biopsy specimens of participants; half were diagnosed with underlying conditions (leukemia, lymphoma).^[10]^ Thus, liver biopsy can help diagnose lymphoma-associated HLH. The guidelines recommend liver biopsy but also acknowledge that findings are often nonspecific. Additionally, a multicenter randomized controlled study involving 2056 patients with focal liver lesions demonstrated that CEUS-GB had an overall diagnostic accuracy of 96%, significantly outperforming US-GB (93%, *P* = .002).^[11]^ Though HLH patients were not included in this study, the advantage of CEUS for the real-time differentiation of active tissue remains applicable, particularly when conventional ultrasound fails to visualize the lesion, as in this case. These findings emphasize the importance of etiological evaluation for secondary HLH through FCM of the liver or lymph nodes.

Given that the patient had an active Epstein–Barr virus (EBV) infection, PET/CT findings highly suggestive of lymphoma, and negative HLH gene mutation screening, we excluded primary HLH and favored a diagnosis of secondary HLH. Flow cytometry is a high-throughput cellular analysis technique based on fluorescent labeling and scattered light signals. It can accurately identify cellular immunophenotypes and quantify apoptotic cells and cytokine levels. For inconclusive pathological results, FCM is accurate and helpful for diagnosing secondary HLH.^[12,13]^

In this case, the bone marrow biopsy pathology failed to identify hematophagocytosis. This differs from the diagnostic pathway recommended in the 2022 Chinese guidelines, which involves confirmation through bone marrow aspiration and histopathology.^[3]^ The possible reasons for this difference are as follows: hemophagocytosis is nonspecific to HLH and can occur in infections, postoperative conditions, and other disorders^[14]^ and bone marrow hematophagocytosis assessment can be subjective. Therefore, we performed CEUS-guided liver biopsy and combined it with FCM analysis. This case study highlights that simply relying on morphological findings may result in the failure to diagnose secondary HLH.^[15,16]^

To compensate for the morphological inadequacy and clarify the lymphoma cell origin, we used flow cytometric immunophenotyping. This can detect a small number of tumor cells from a massive necrotic hepatic biopsy sample. The FCM results showed an abnormal cell population with moderate side scatter (SSC) and high forward scatter, suggestive of mononuclear macrophages or abnormal lymphoma cells. These cells did not express CD19 or CD20 but highly expressed CD56, CD2, and CD7, implying that they were NK/T-cells rather than B-cells. Additionally, FCM can assist in the preliminary screening for primary and secondary HLH by detecting perforin expression and granule release.^[17,18]^ In this case, since the clinical presentation and related tests were clearly suggestive of secondary HLH, assays for perforin expression and granule release tests were not performed.

This study has several limitations that should be acknowledged. First, this is a case report of a single case, and thus, statistical analysis was not performed. Second, sampling bias may have occurred due to tumor heterogeneity, necrosis sampling, and cell damage during FCM preparation. Third, patients with severe coagulation disorders cannot tolerate needle biopsy^[3]^; thus, this approach may not be generalizable to other patients.

## 4. Conclusion

In summary, this report presents a case of CEUS-guided liver puncture biopsy combined with FCM analysis to diagnose hemophagocytic syndrome secondary to NK/T-cell lymphoma. This case not only demonstrates the precision and minimal invasiveness of CEUS guidance compared with ordinary US-GB but also highlights the value of FCM compared with conventional pathology in lymphoma diagnosis. We hope that this case study will provide a reference and learning for fellow physicians.

## Acknowledgments

The authors extend their sincere appreciation to the patient for her participation, which made this work possible. Informed consent from the patient has been obtained for publication of the case.

## Author contributions

**Conceptualization:** Shuyuan Tian.

**Data curation:** Shuyuan Tian.

**Formal analysis:** Huihui Ma, Qing Pan, Xin Jin, Shuyuan Tian.

**Writing – original draft:** Huihui Ma, Shuyuan Tian.
